# Tailoring *Escherichia coli* BL21 (DE3) for preferential xylose utilization via metabolic and regulatory engineering

**DOI:** 10.1007/s00253-025-13430-4

**Published:** 2025-02-28

**Authors:** Eliseo R. Molina-Vázquez, Luis Caspeta, Guillermo Gosset, Alfredo Martínez

**Affiliations:** Departamento de Ingeniería Celular y Biocatálisis, Instituto de Biotecnología, Universidad Nacional Autónoma de México, Av. Universidad 2001, Col. Chamilpa, 62210 Cuernavaca, Morelos Mexico

**Keywords:** *Escherichia coli*, Catabolite repression, Xylose, Xylose-selective strain, Sugar co-consumption

## Abstract

**Abstract:**

Xylose is the most abundant pentose in nature. However, it is usually obtained in mixtures with glucose, leading to carbon catabolite repression in many microorganisms. Among *E. coli* lineages, significant metabolic and regulatory differences exist, requiring distinct metabolic engineering strategies to develop a xylose-selective phenotype in the strains W, K-12, and C. In this study, strain ES02 was engineered from *Escherichia coli* BL21 (DE3) as a xylose-selective strain by deleting the *glk*, *ptsG*, and *manZ* genes*.* However, when grown in a mixture of xylose and glucose, this strain’s specific growth rate and xylose consumption rate decreased by about 50% compared to cultures with only xylose. A modified version of the xylose-responsive transcriptional activator XylR^Q31K^ was utilized to overcome this issue. The resulting strain ES04 (BL21 (DE3) *Δglk*, *ΔmanZ*, *ΔptsG*, *xylR::Km*^*r*^, *lacZ::xylR*^*C91A*^*-Gm*^*r*^) efficiently used xylose as carbon source either alone or in a mixture with glucose, with a specific xylose consumption rate 75% higher than that of the wild-type strain BL21(DE3). Unexpectedly, strain ES04 partially recovers the ability to grow and consume glucose at a low rate, preferentially consuming xylose over glucose in sugar mixtures, revealing an altered carbon catabolite repression phenotype. Transcriptomics analysis suggested that glucose assimilation in this strain was related to the overexpression of the galactitol operon *gatDCBAZY.* Further inactivation of this operon confirmed its participation in glucose assimilation.

**Key points:**

*• XylR* ^*Q31K*^ *alleviates carbon catabolite repression in the xylose-selective strain ES04.*

*• Galactitol operon overexpression in ES04 links to partial glucose utilization.*

*• ES04 strain preferentially uses xylose over glucose, revealing altered CCR.*

**Supplementary Information:**

The online version contains supplementary material available at 10.1007/s00253-025-13430-4.

## Introduction

*Escherichia coli* is extensively studied at molecular, physiological, and metabolic levels. The lineages K-12, B, C, and W are commonly used for research and biotechnology. Despite their highly similar genome sequences and organization, their metabolism and molecular regulation differ (Hayashi et al. 2006; Studier et al. 2009). For example, in cultures with glucose, strain BL21’s specific growth rate decreases by 60% comparing aerobic to anaerobic conditions, while strain W maintains a similar rate in both conditions due to a threefold increase in glucose consumption. Additionally, strain W can efficiently assimilate sucrose and cellobiose, unlike *E. coli* B, C, and K-12 (Archer et al. 2011; Monk et al. 2016). In turn, BL21 lacks Lon and OmpT proteases and possesses an active type II secretion system, making it the workhorse for recombinant protein production (Studier et al. 2009; Yoon et al. 2012). BL21 produces less acetate than other *E. coli* strains because of higher activity of the glyoxylate shunt, a decreased expression of acetate formation pathways, and increased acetate assimilation (Phue et al. 2005; Yoon et al. 2012; Monk et al. 2016). Additionally, BL21 has a reduced flux in the pentose phosphate pathway (PPP) due to the lack of 6-phosphogluconolactonase (*pgl*) (Monk et al. 2016). These differences resemble the dissimilarities between the strains of *E. coli* and the distinctive architecture of their regulatory networks, which should be considered when developing engineered strains for bioprocessing.

**Fig. 1:**
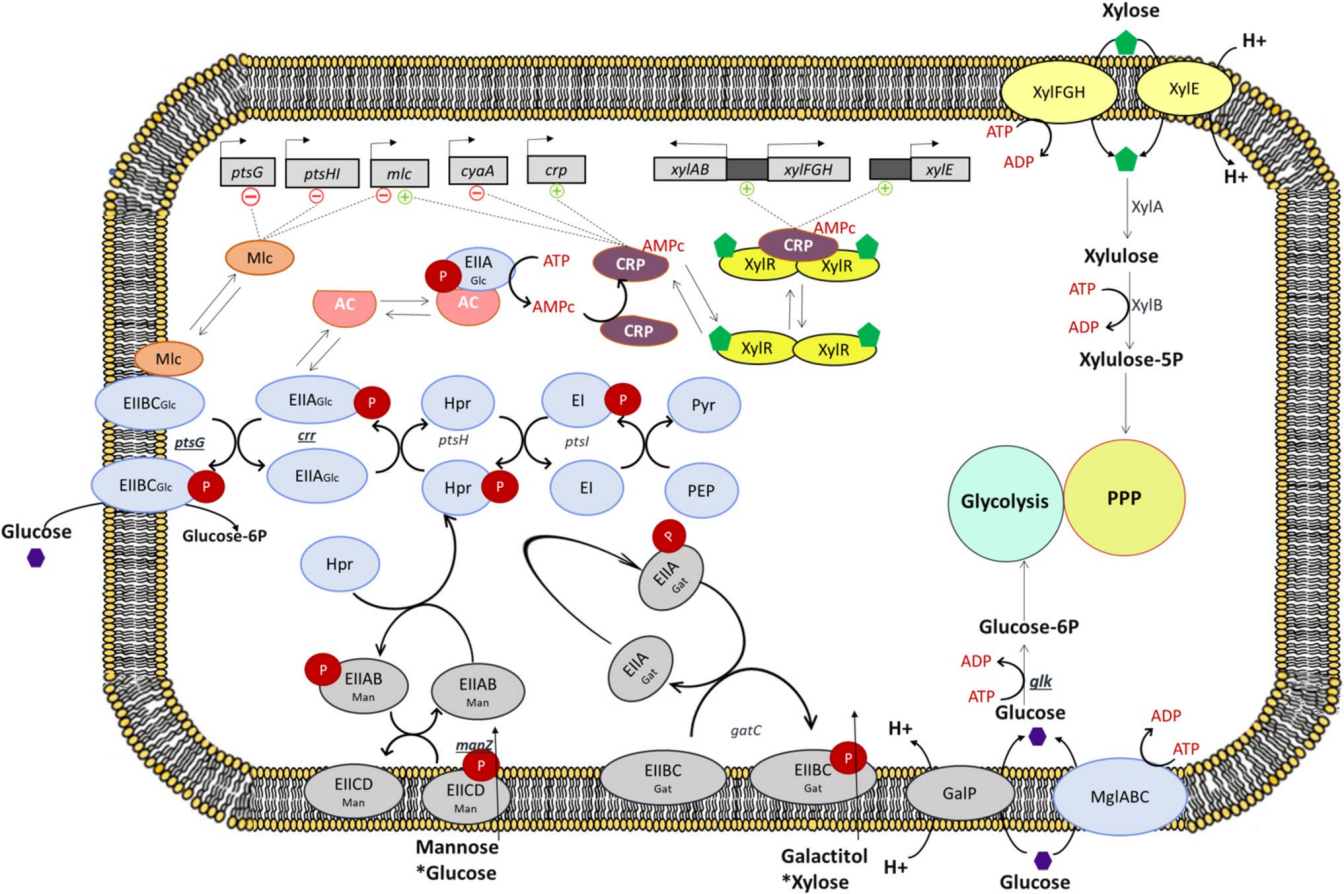
Catabolic pathways and regulation nodes for glucose and xylose assimilation. In a xylose-glucose mixture, *E. coli* WT strains use the general PTS system (*crr*, *ptsH*, *ptsI*) through the glucose-specific PTS-dependent permease EIIBC^Glc^ (*ptsG*) for glucose assimilation. When glucose is depleted, components EIIA^Glc^ and EIIB^Glc^ are phosphorylated, and EIIA.^Glc^ ~ P binds the enzyme adenylate cyclase (AC), promoting cAMP synthesis. High levels of cAMP activate CRP, forming the CRP-cAMP complex. The start of the transcription of the loci responsible for xylose transportation and catabolism requires that the CRP-cAMP complex recognizes specific DNA sequences known as CRP-binding sites located upstream of the promoter regions of *xylE*, *xylFGH*, and *xylAB*. This binding causes a conformational change in the DNA, making these promoter regions available and promoting the binding of an active XylR complex. This active complex consists of a dimmer of XylR, binding a xylose molecule each (xyl-XylR-XylR-xyl). The full activation of xylose metabolism could be achieved by the expression of XylR variants that have an increased affinity to the promoter regions, even in the absence of CRP-cAMP complex (Deutscher et al. 2006; Groff et al. 2012; Sievert et al. 2017; Choudhury et al. 2018; Heo et al. 2021). * Means unspecific sugar transport. Created in BioRender. Castrejon, S. (2024) BioRender.com/n11p797

The rising interest in developing metabolic engineering strategies for xylose as a carbon source is due to its abundance in nature. However, xylose is often obtained in a mixture with glucose, leading to consumption limited by carbon catabolite repression (CCR) and diauxic growth. In glucose-xylose mixtures, *E. coli* uses the phosphotransferase system (PTS) for glucose import and phosphorylation (Fig. 1). When glucose is depleted, components EIIA^Glc^ (*crr*) and EIIB^Glc^ (*ptsG*) are phosphorylated, promoting the formation of CRP-cAMP complex. This complex recognizes and binds specific DNA sequences known as CRP-binding sites located upstream of the promoter regions of *xylE*, *xylFGH*, and *xylAB*. This binding causes a conformational change in the DNA, making these promoter regions available and promoting the binding of an active XylR complex (a dimer of XylR, each one binding a xylose molecule) (Deutscher et al. 2006; Groff et al. 2012; Choudhury et al. 2018; Barthe et al. 2020). Xylose uptake is mediated through XylFGH (an ABC transporter) and XylE (a symporter). Once in the cytoplasm, xylose is isomerized to xylulose by XylA, phosphorylated to xylulose-5-phosphate by XylB, and then metabolized through the PPP (Song and Park 1997).

Diverse approaches have been used to develop *E. coli* strains for simultaneous xylose-glucose consumption (Monk et al. 2016), including adaptive laboratory evolution (ALE) and direct metabolic engineering (Cirino et al. 2006; Utrilla et al. 2012; Sievert et al. 2017; An et al. 2021; Heo et al. 2021).

The ALE experiment in a K-12 strain led to the discovery of non-canonical transporter GatC^S184L^, which increases the growth rate and the specific xylose consumption rate by 1.5 and twofold, respectively, under non-aerated conditions (Utrilla et al. 2012). While ALE in BL21 (DE3), C and W lineages resulted in the discovery of xylose activator variants, XylR^Q31K^, XylR^R121C^, and XylR^P363S^, respectively. All these variants allowed the co-consumption of xylose and glucose (Sievert et al. 2017; Heo et al. 2021).

Another successful approach has been the co-culture of *E. coli* strains free of CCR, which specialize in assimilating a specific carbon source, creating glucose-selective strains by eliminating *xylA* or *xylR* (Eiteman et al. 2008; Xia et al. 2012; Flores et al. 2020). However, developing xylose-selective strains has been challenging due to their plasticity for glucose assimilation and tight CCR regulation. In the case of K-12, a xylose-selective strain was created by disrupting the glucokinase (*glk*) and the main glucose and mannose PTS components EIIB^Glc^ (*ptsG*) and EIIB^Man^ (*manZ*) (Curtis and Epstein 1975; Eiteman et al. 2008). For *E. coli* C, achieving this phenotype required the additional deletion of EIIA^Glc^ (*crr*) (Xia et al. 2012). Similarly, the xylose-selective *E. coli* W strain was obtained by eliminating *ptsI*, *ptsG*, *galP*, and *glk*, and replacing native XylR with the variant XylR^R121C P363S^ (Flores et al. 2020).

*E. coli* strains C, K-12, and W exhibit notable genomic, metabolic, and regulatory differences, necessitating strain-specific metabolic engineering strategies for developing a xylose-selective phenotype. However, previous research has primarily focused on the resulting phenotype and its application in sugar mixture co-consumption, with little discussion about the distinctive genetic strategies employed and the physiological changes observed when the selective strains are grown in xylose or mixed sugars. This work aimed to develop a xylose-selective strain derived from *E. coli* BL21 (DE3) and understand its growth and xylose and glucose assimilation patterns in single or mixed sugars.

## Materials and methods

### Strains and plasmids

The strains of *E. coli* and plasmids used in this study are described in Table 1, while the list of primers is available in the supplementary material (Table S1). The xylose-selective strain ES02 (BL21 (DE3) Δ*glk*, Δ*manZ*, Δ*ptsG*) was constructed inactivating *ptsG*, *manZ*, and *glk* genes using phage P1 transduction system with the respective strains from the Keio collection as previously reported (Baba et al. 2006). After each gene inactivation, the kanamycin resistance cassette was removed using the pCP20 plasmid, as reported elsewhere (Datsenko and Wanner 2000). The strain ES03 (ES02 Δ*crr*) was constructed by inactivating the *crr* gene from ES02 following the lambda red system protocol as previously reported (Datsenko and Wanner 2000), the donor molecule for the recombination was directly amplified by PCR from the genomic DNA of the strain Keio Δ*crr*. The native *xylR* gene was eliminated from strain ES02 following a similar protocol with genomic DNA from the strain Keio Δ*xylR*, and the intermediate strain ES02 *xylR::Km*^*r*^ was obtained. In this strain, *lacZ* was replaced with the *xylR*^C91A^ (XylR^Q31K^) variant following a protocol previously reported (Sabido et al. 2013), resulting in the strain ES04 (ES02 *xylR::Km*^*r*^, *lacZ::xylR*^*C91A*^*-Gm*^*r*^). Subsequently, the *gatC* gene and the *gatDCBAZY* operon were deleted following the lambda red system protocol, resulting in the strains ES05 (ES04 Δ*gatC*) and ES06 (ES04 Δ*gatDCBAZY*), respectively.

**Table 1:** Strains and plasmids used in this study

Strains	Genotype	Reference	
BL21 (DE3)	*E. coli* str. B F^–^ *ompT gal* *dcm lon hsdS*_*B*_(*r*_*B*_^–^*m*_*B*_^–^) λ(DE3 [*lacI lacUV5 T7p07 ind1 sam7 nin5*]) [*malB*^+^]_K_^−1^_2_(λ^S^)	(Studier and Moffatt 1986)	
ES02	BL21 (DE3) Δ*glk*, Δ*manZ*, Δ*ptsG*	This study	
ES03	ES02 Δ*crr*	This study	
ES04	ES02 ES02 *xylR::Km*^*r*^, *lacZ::xylR*^*C91A*^*-Gm*^*r*^	This study	
ES05	ES04 *gatC::Km*^*r*^	This study	
ES06	ES04 *gatDCBAZY::Km*^*r*^	This study	
Plasmids			
pKD46	Confers carbenicillin resistance and expresses the λ-red phage recombinases (β, γ, and Exo). Its origin of replication is temperature sensitive	(Datsenko and Wanner 2000)	
pCP20	Confers carbenicillin and chloramphenicol resistance and expresses the flippase for the FRT flanked antibiotic cassette removal. Its origin of replication is temperature sensitive	(Datsenko and Wanner 2000)	
pCL_GatC^S184L R216C^	*gatC*^*C551T, C646T*^ cloned into the low copy number pCL1920	This study	
pCL_XylR^Q31K^	*xylR*^C91A^ cloned into the low copy number pCL1920		This study

XylR^Q31K^ (*xylR*^C91A^) was constructed by overlapping extension PCR. The region from 1 to 106 pb was amplified with the primers F1_xylR and R1_mutxylR, and then, the second fragment from 71 to 1201 pb was amplified with primers F2_mutxylR and R2_xylR. The point mutation C91A was introduced in the overhangs of both fragments in the region from 71 to 106 pb, the same that was used as a homology for fusing the PCR products. In addition, the primers F1xylR and R2xylR introduced overhangs with homology to the pCL1920 vector, which was linearized with the primers FlinpCL and RlinpCL. The XylR^Q31K^ was cloned into the pCL1920 linearized vector by the circular polymerase extension cloning method (Quan and Tian 2011). The pCL_GatC^S184L R216C^ plasmid was obtained from a mutagenesis library previously constructed in our laboratory (unpublished data). GatC^S184L R216C^ (encoded by *gatC*^*C551T, C646T*^) is a double protein mutant derived from GatC^S184L^ reported to improve xylose consumption in a strain derived from K-12 (Utrilla et al. 2012).

### Culture conditions

The strains were grown on LB or minimal medium M9 supplemented with 20 mM MOPS and 33 mM xylose or 28 mM glucose or a mixture of 16.5 mM xylose with 14 mM glucose. The cultures were carried out in 250-mL flasks with 50-mL medium at 300 rpm and 37 °C for the growth kinetics. When necessary, the appropriate antibiotic was supplemented (Cb at 100 µg mL^−1^, Km at 30 µg mL^−1^, or Gm at 10 µg mL^−1^). The genes cloned into the plasmids derived from pCL1920 were induced with 60 µM IPTG. All the inocula for the experiments were developed in M9 medium with xylose 33 mM for pre-adaptation. Samples were collected every two hours during the exponential phase (12 h) and then every 4 h until sugar depletion. The preliminary evaluation of ES02 and ES03, growth kinetics performance, was conducted using half of the carbon source concentrations. These results, used to determine the most suitable strain for further research, are included in the supplementary material.

### Analytical procedures

Growth was spectrophotometrically monitored as optical density (OD) at 600 nm and converted to dry cell weight per liter using the factor 1 OD = 0.43 g_DCW_ L^−1^. The samples were centrifuged at 12,000 rpm for 4 min, and the supernatants were stored at − 20 °C until analysis. Glucose and xylose were determined by high-pressure liquid chromatography (Waters U6K, Millipore Co., Milford, MA, USA) using an Aminex HPX-87H ion exclusion column (300 × 7.8 mm; Bio-Rad Laboratories, Hercules, CA), 5.0 mM H_2_SO_4_ solution as the mobile phase in isocratic mode at 0.5 mL mL^−1^ at 50 °C, and using a refractive index detector (Model 2410, Waters, Millipore Co., Milford, MA, USA).

### Statistical analysis

The experiments were performed in triplicate. To determine the differences in the specific growth rate and specific sugar uptake rate among the different strains of *E. coli* developed in this study, statistical analyses were conducted using GraphPad Prism software. Brown–Forsythe test was used to ensure the homogeneity of variances between the groups, validating the data, so it could be subjected to analysis of variance (ANOVA). A one-way ANOVA was conducted to compare the means of µ or qs for the different groups (strains). Tukey’s multiple comparisons test was applied to determine significant differences between specific pairs of strains. The adjusted *p* values and confidence intervals for the differences in means were calculated with an alpha level of 0.05.

### Transcriptomics analysis

As previously described, BL21 (DE3) and ES04 strains were grown in glucose or xylose. When strains reached the middle exponential phase (~ 0.3–0.4 g_DCW_ L^−1^), 10 mL of the culture were mixed with 500 µL of RNAlater™ (Thermo Fisher Scientific, Baltics, UAB), then gently mixed by inversion. The samples were centrifuged at 5000 rpm for 5 min, the supernatant was discarded, and the pellet was frozen at − 70 °C until further analysis. Total RNA was isolated using an RNAeasy Midi kit (cat. no. 75142, Qiagen, Hilden, Germany). Chromosomal DNA was removed with the turbo-DNA free kit (cat no. AM1907, Thermo Fisher Scientific, Baltics, UAB). The samples were analyzed at the DNA Microarrays Unit of the Institute of Cellular Physiology at the Universidad Nacional Autónoma de Mexico. cDNA was synthesized using mRNA as a template, followed by cDNA staining and hybridization in DNA microarray plates (Miller et al. 2002). A partial analysis of gene expression changes was also performed at the DNA Microarrays Unit.

The raw data from the microarray analysis were deposited in the NCBI database under the accession number GPL34929. Transcriptional profiles of strain ES04 with respect to BL21 (DE3) grown on xylose or glucose were compared. Differentially expressed genes were selected by calculating an intensity‐­ dependent Z score as zi = (Ri − mean(*R*))/(sd(*R*)), where zi is the z score for each element gene, Ri is the log ratio for each element gene, and sd(*R*) is the standard deviation of the log ­ratio. Genes with |*z* score|> 2.5 standard deviations were considered significantly expressed. The ClusterProfiler package was used to perform gene enrichment analysis (GEA), and *p* value and *q* value were adjusted by the HB method with a cutoff value < 0.05 (Wu et al. 2021).

## Results

### Constructing xylose-selective strains from* E. coli* BL21 (DE3)

A previous study based on multi-locus sequence typifying had shown that strain BL21 (DE3) is closer to K-12 and C than W (Monk et al. 2016). Therefore, the metabolic engineering strategies previously reported for K-12 and C were applied to generate xylose-selective strains from BL21 (DE3). The genes *glk*, *manZ*, and *ptsG* were inactivated, resulting in the strain ES02 (BL21 (DE3) Δ*glk*, Δ*manZ*, Δ*ptsG*). Then, the *crr* gene was eliminated to generate strain ES03 (ES02 Δ*crr*).

Both strains were evaluated in flask cultures with M9 medium supplemented with 16 mM xylose, 14 mM glucose, or a mixture of 8 mM xylose and 7 mM glucose (Fig. S1). Both strains could not efficiently use glucose as a carbon source, consuming barely 1.8 mM in 24 h. Under similar conditions, the WT BL21 (DE3) depleted 28 mM glucose in 10 h. The negligible glucose consumption observed in ES02 and ES03 is consistent with reports for other xylose-selective strains (Eiteman et al. 2008, 2009; Xia et al. 2012; Flores et al. 2020). When comparing the growth of these strains on xylose, ES02 showed superior performance, depleting 12 mM of this sugar in 20 h, whereas the ES03 strain barely consumed 7 mM in 24 h. In the xylose-glucose mixture, ES02 depleted the 7 mM xylose even in the presence of glucose, although it took around 24 h. Surprisingly, under the same conditions, ES03 only consumed 1.2 mM xylose in 24 h, displaying a different phenotype than that previously reported for strain C with the same genotype (Xia et al. 2012). The decrease in xylose consumption in the mixture suggested that glucose interfered with xylose transport or catabolism. Since ES02 showed limited glucose consumption and a higher xylose consumption rate than ES03 (4.73 vs. 2.23 mmol_xyl_ g_DCW_^−1^ h^−1^), the strain ES02 was selected for further studies.

### Differential xylose utilization between the strains ES02 and BL21 (DE3)

**Fig. 2:**
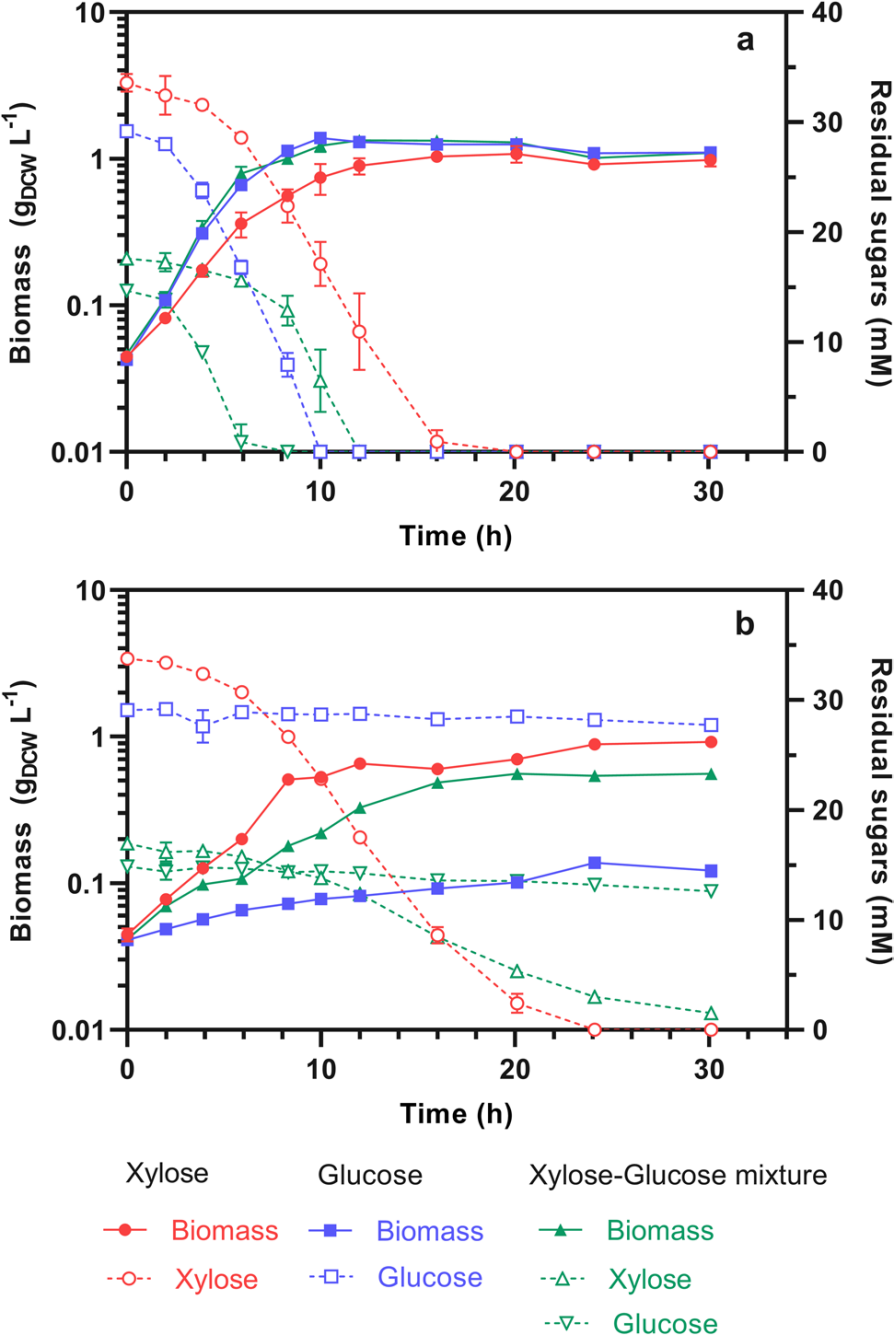
Growth and residual sugars kinetics for the xylose-selective strains. **a** BL21 (DE3), **b** ES02 (BL21 DE3 Δ*glk*, Δ*manZ*, Δ*ptsG*) in defined M9 medium with 33 mM xylose (circles) or 28 mM glucose (squares), or a mixture (triangles) of 16.5 mM xylose with 14 mM glucose. Solid lines and filled shapes indicate biomass, whereas dotted lines and hollow shapes indicate residual sugars. In the sugar mixture, straight triangles indicate xylose and inverted triangles indicate glucose

In cultures containing xylose (as shown in Figs. 2 and  5), the strains ES02 and BL21 (DE3) reached the same specific growth rate (µ_xyl_) of 0.3 h^−1^. However, the specific xylose consumption rate (qs_xyl_) of ES02 was 28% lower (*p* > 0.05), decreasing from 6.45 to 4.65 mmol_xyl_ g_DCW_^−1^ h^−1^.

In the xylose-glucose mixture, the strain BL21 (DE3) first depleted glucose (~ 6 h), and only then, it consumed xylose. In contrast, ES02 did not consume glucose, growing exclusively on xylose. As expected, BL21 (DE3) grew faster than ES02 (0.35 vs. 0.14 h^−1^) because BL21 (DE3) used glucose as the primary carbon source, which agrees with previous reports that have demonstrated that glucose rather than xylose leads to a greater growth rate in different *E. coli* strains, including BL21 (DE3) (Heo et al. 2021), BW21135 (Gonzalez et al. 2017), and C (Xia et al. 2012).

Notably, ES02 experienced a 55% reduction (*p* > 0.05) in the µ_xyl_ from 0.30 to 0.14 h^−1^ and 45% in qs_xyl_ from 4.66 to 2.55 mmol_xyl_ g_DCW_^−1^ h^−1^ in culture with xylose-glucose mixture regarding solely xylose. A similar reduction in µ_xyl_, from 0.72 to 0.55 h^−1^, was reported for a xylose-selective *E. coli* C strain under analogous conditions (Xia et al. 2012).

To decipher the cause of the decrease in xylose consumption when the strain ES02 was cultured in the xylose-glucose mixture, a non-canonical xylose transporter and a mutated version of the transcriptional activator XylR were evaluated. For the first approach, if the consumption rate was associated with a reduction in xylose transport, the expression of the non-canonical transporter GatC^S184L R216C^ should increase qs_xyl_. Alternatively, if the phenotype observed in ES02 followed a regulatory process, the expression of a variant of XylR^Q31K^, which is insensitive to the intracellular levels of cAMP, would improve the xylose metabolism independently of the presence of glucose (Heo et al. 2021).

### Protein XylR^Q31K^ in strain ES02 enhanced xylose consumption

**Fig. 3:**
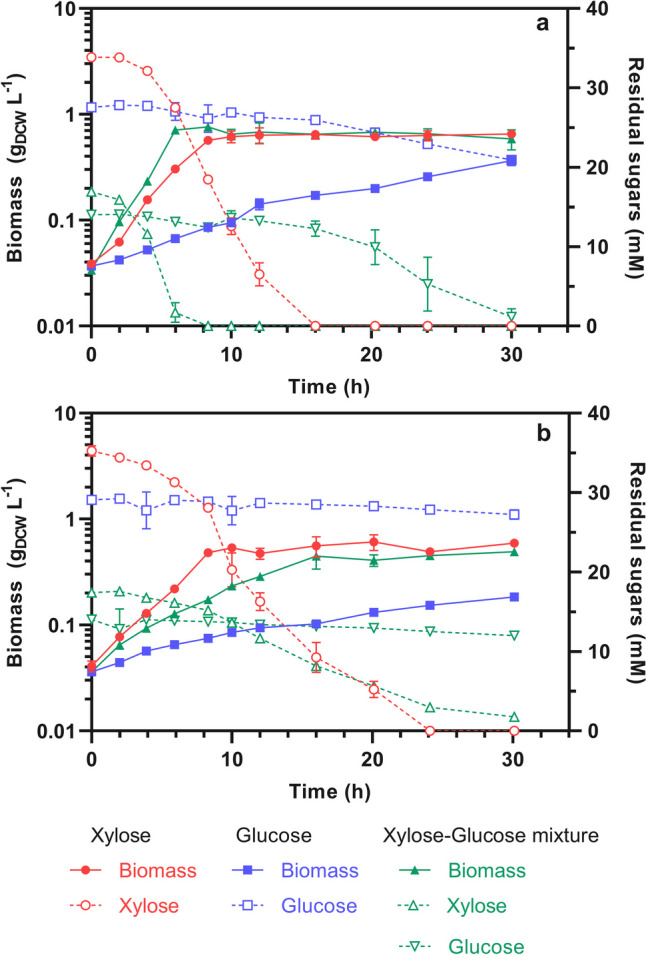
Growth and residual sugars kinetics of the strains. **a** ES02/pCL_XylR^Q31K^ and **b** ES02/pCL_GatC^S184L R216C^ in defined M9 medium with 33 mM xylose (circles) or 28 mM glucose (squares), or a mixture (triangles) of 16.5 mM xylose with 14 mM glucose. Solid lines and filled shapes indicate biomass, whereas dotted lines and hollow shapes indicate residual sugars. In the sugar mixture, straight triangles indicate xylose and inverted triangles indicate glucose

**Fig. 4:**
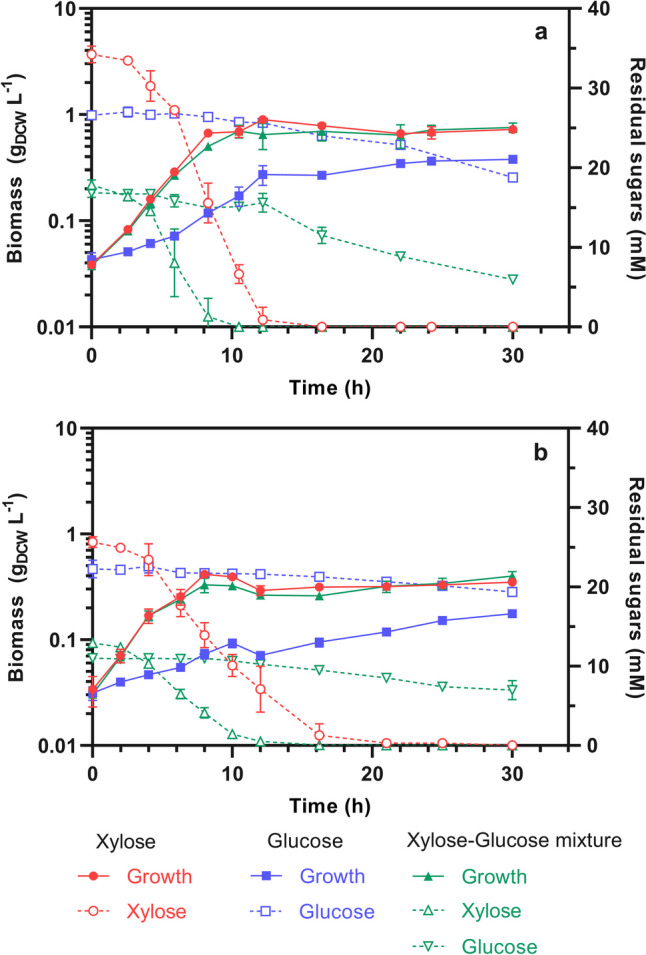
Growth and residual sugars kinetics of the strains. **a** ES04 (ES02 *xylR*::*Km*^r^, *lacZ*::*ylR*^*C91A*)^) and **b** ES06 (ES02 Δ*xylR*, *lacZ*::*xylR*^*C91A*^, *gatDCBAZY*::*Km*^*r*^) in defined M9 medium with 33 mM xylose (circles) or 28 mM glucose (squares), or a mixture (triangles) of 16.5 mM xylose with 14 mM glucose. Solid lines and filled shapes indicate biomass, whereas dotted lines and hollow shapes indicate residual sugars. In the sugar mixture, straight triangles indicate xylose and inverted triangles indicate glucose

GatC^S184L R216C^ and XylR^Q31K^ protein variants were independently expressed in the strain ES02 using the low copy number plasmid pCL1920 (Table 1). As observed in Fig. 3B, the protein GatC^S184L R216C^ in ES02 did not significantly (*p* > 0.05) improve the µ_xyl_ (0.3 vs. 0.3 h^−1^) or the qs_xyl_ (4.66 vs. 4.82 mmol_xyl_ g_DCW_^−1^ h^−1^) in cultures with either xylose or the mixture xylose-glucose. In contrast, the expression of XylR^Q31K^ significantly enhanced the performance of the strain ES02. When the ES02/pCL_XylR^Q31K^ strain was grown with xylose, no significant change (*p* > 0.05) in the µ_xyl_ was observed (0.30 to 0.34 h^−1^). In contrast, a 2.25-fold increase (*p* > 0.05) in qs_xyl_, from 4.66 to 10.51 mmol_xyl_ g_DCW_^−1^ h^−1^ was observed. Similarly, in the xylose-glucose mixture, the strain ES02/pCL_XylR^Q31K^ outperformed ES02, increasing not only the qs_xyl_ by 3.6-fold, from 2.55 to 9.16 mmol_xyl_ g_DCW_^−1^ h^−1^, but also the µ_xyl_ by 2.7-fold, from 0.14 to 0.38 h^−1^ (Fig. 4A).

XylR^Q31K^ improved xylose consumption in ES02 and alleviated the apparent repression caused by the presence of glucose in cultures with a xylose-glucose mixture. As a result, the strain ES02/pCL_XylR^Q31K^ depleted the 17 mM xylose present in the xylose-glucose mixture in less than 8 h, whereas the strain ES02 required more than 30 h.

### Chromosomal integration of *xylR*^C91A^

Since the episomal expression of gene *xylR*^C91A^ that encodes XylR^Q31K^ resulted in an efficient strain for consuming xylose in the presence of glucose, the strain ES02 was further engineered. The wild-type *xylR* gene was replaced with a kanamycin resistance cassette, and the *lacZ* gene was replaced by *xylR*^C91A^, resulting in the strain ES04 (ES02 *xylR::Km*^*r*^, *lacZ::xylR*^C91A^). The gene *xylR*^C91A^ was integrated under the control of the *lac* operator and the strong promoter *trc* to maintain the same expression level of ES02/pCL_XylR^Q31K^.

**Fig. 5:**
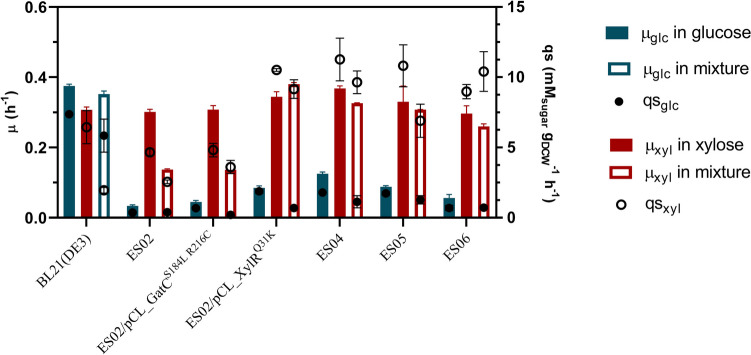
The specific growth and sugar consumption rates of the xylose-selective strains derived from *E. coli* BL21 (DE3) are presented. The bars, representing specific growth rates (left axis), show blue bars for cultures with glucose, red bars for cultures with xylose, and white bars for cultures with the mixture of xylose-glucose. The contour color in the white bars indicates the preference for carbon source usage in the mixture of xylose-glucose, with blue for glucose and red for xylose. The circles, representing the specific sugar consumption rate (right axis), are differentiated with black circles for glucose and white circles for xylose

The comparison between strain ES04 and the previous strain ES02 indicates no significant change (*p* > 0.05) in the µ_xyl_ (0.37 vs. 0.35 h^−1^) or the qs_xyl_ (10.51 vs. 11.26 mmol_xyl_ gDCW^−1^ h^−1^) when cultured with xylose (see Fig. 5). However, ES04 could metabolize 34 mM xylose in 12 h, whereas ES02/pCL_XylRQ31K required 16 h. This improvement is directly related to an increase in the maximum biomass produced (*X*_max_) from 0.58 to 0.85 gDCW L^−1^. This increase in *X*_max_ could be linked to a reduction in the metabolic burden experienced by ES04, which does not carry a plasmid. Lastly, the µ_xyl_ of ES04 was 0.37 h^−1^, the same as the µ_glc_ of the BL21(DE3), marking an important accomplishment for a xylose-selective strain.

### XylR^Q31K^ partially recovers glucose consumption in the xylose-selective strains

The ES02/pCL_XylR^Q31K^ and ES04 strains showed improved µ_xyl_ and qs_xyl_ in cultures with xylose or the xylose-glucose mixture (Fig. 5). However, an unexpected side effect was identified in cultures with glucose. Both strains partially recovered their glucose consumption ability (see empty squares in Figs. 3A and 4A). Moreover, this glucose consumption was very slow, with around 8 mM consumed in 30 h. Likewise, glucose consumption was also detected in cultures with the xylose-glucose mixture; however, glucose was not consumed while xylose was available. Once xylose was depleted (*t* = 8), glucose consumption slowly began after a lag phase of 4 h. The glucose consumption rate then slightly increased, supporting the utilization of approximately 8–12 mM glucose in 18 h.

This behavior indicates that the presence of xylose impedes glucose utilization. When this pentose is depleted, a lag phase is observed, and only then is effective glucose consumption initiated. These findings suggest that xylose represses glucose consumption in ES02/pCL_XylR^Q31K^ and ES04, revealing an altered CCR mechanism where xylose is preferentially consumed over glucose.

### Transcriptomics revealed overexpression of the galactitol operon in ES04 grown on glucose

The transcriptional profiles of the xylose-selective ES04 strain were compared to those from the parental strain BL21 (DE3). The experiments were performed in an M9 medium with glucose or xylose as carbon sources, resulting in 170 and 209 differential expressed genes (|*z* score|> 2), respectively. Gene set enrichment analysis (GSEA) revealed no significant differential expression between ES04 and BL21 grown on xylose. However, key individual genes displayed different expression profiles (Fig. 4). In contrast, the GSEA of the transcriptomic response of ES04 compared to BL21 grown in glucose, with statistical significance determined by an adjusted *p* value < 0.05 after correction for multiple testing using the Benjamini–Hochberg (BH) method (Figs. S3 and S4), revealed that the operons responsible for the transportation (*xylE*) and metabolism of xylose (*xylA* and *xylB*) were upregulated (> twofold). Unexpectedly, the galactitol operon *gatDCBAZY* was also significantly overexpressed (> twofold).

### The inactivation of the galactitol operon reduced glucose consumption in strain ES04

The galactitol operon is composed of genes encoding the metabolic enzymes GatD (*gatD*), GatZ (*gatZ*), and GatY (*gatY*), the permease GatC (*gatC*), and two PTS components EIIA (*gatA*) and EIIB (*gatB*) (Nobelmann and Lengeler 1996; Shakeri-Garakani et al. 2004)*.* According to transcriptomics analysis, some of these elements or their combined activities might be responsible for glucose consumption in the strain ES04. Gene *gatC* was inactivated to determine if the permease GatC could internalize glucose, *g*iving rise to the strain ES05 (ES04 *ΔgatC*). Cultures of this strain in glucose, xylose, or the xylose-glucose mixture (Figs. 5 and S2) resulted in no significant µ or qs difference (*p* < 0.05) when compared to ES04, showing that GatC was not mediating the internalization of glucose.

The next approach was to inactivate the entire *gatDCBAZY* operon, resulting in the strain ES06 (ES04 Δ*gatDCBAZY*). The strain ES06 in glucose exhibited a significant reduction (*p* < 0.05) of 53% in the µ_glc_ and 61% in the qs_glc_ compared to ES04 (Fig. 5). The µ_glc_ decreased from 0.13 to 0.06 h^−1^, and the qs_glc_ from 1.8 to 0.7 mmol_glc_ g_DCW_^−1^ h^−1^. Similar results were obtained in the xylose-glucose mixture, where qs_glc_ decreased by 36% from 1.14 to 0.72 mmol_glc_ g_DCW_^−1^ h^−1^.

While inactivation of the *gatDCBAZY* significantly decreased glucose consumption, a complete reversion of this phenotype was not observed, indicating that this operon was partially responsible for glucose assimilation in ES04. Moreover, this inactivation brought a side effect, with a 50% reduction in the maximum biomass achieved by the strain ES06 in xylose, glucose, or xylose-glucose mixture (Fig. 4B).

## Discussion

### Previous strategies for xylose-selectivity resulted in unexpected phenotypes of BL21 (DE3)

The inactivation of *glk*, *manZ*, and *ptsG* in the BL21 background (strain ES02) resulted in negligible glucose consumption, which agrees with previous reports (Curtis and Epstein 1975; Eiteman et al. 2008). Conversely, the strain ES02 growing on xylose retained the same µ_xyl_ as BL21(DE3) but showed a 28% decrease in qs_xyl_. Additionally, ES02 showed a 60% reduction in qs_xyl_ in the mixture of xylose-glucose compared to sole xylose, differing from the phenotype observed in other xylose-selective strains derived from K-12, where xylose consumption is unaffected, even in the presence of glucose (Eiteman et al. 2008).

Probably in the xylose-selective strains derived from K-12, the internalization of xylose is facilitated by the symporter XylE and the ABC transporter XylFGH. In contrast, in BL21 (DE3), *xylG* has a single nucleotide deletion (c.1094delC) in the open reading frame (Heo et al. 2021). This frameshift changes the amino acid sequence from residue 365 to residue 405, where a premature stop codon is introduced, resulting in a truncated version of XylG of 404 amino acids. In comparison, the full-length version consists of 513 amino acids. As a result, in the strain ES02, the ABC transporter XylFGH might not be active, and xylose might primarily be channeled via the XylE symporter (Zhu et al. 2022), explaining the decrement in qs_xyl_ observed in ES02 growing in the xylose-glucose mixture. It has been reported that XylE can recognize and bind glucose but not transport it, demonstrating that this hexose can act as a competitive inhibitor with a Ki of 3.2 mM (Madej et al. 2014). When culturing strain ES02 in the xylose-glucose mixture, glucose may compete for the recognition site of the XylE symporter, consequently reducing the xylose consumption rate.

Another possibility is that despite the inability of ES02 to use glucose, this sugar still triggers a regulation mechanism that decreases the cAMP levels, reducing the availability of the cAMP-CRP complex, and shutting down the xylose catabolic pathways. It has been previously shown that the cAMP-CRP complex is required to activate xylose metabolism (Song and Park 1997; Belliveau et al. 2018). In this case, the inactivation of the *crr* gene (EIIA^glc^) may alleviate the repression exerted by glucose in a mixture with xylose, as demonstrated by the individual mutation of this gene in the strain W3110 (Liang et al. 2015) and integrated into the genotype of a xylose-selective strain derived from *E. coli C* (Δ*crr*, Δ*glk*, Δ*manZ*, Δ*ptsG*, Δ*araA*) (Xia et al. 2012). However, this strategy applied in the BL21 (DE3) background, led to deficient xylose assimilation in ES03 (Δ*crr*, Δ*glk*, Δ*manZ*, Δ*ptsG*), either when xylose was the sole carbon source and more severely affected in the mixture with glucose (Supplementary Fig. S1).

Protein Crr has a major role in the regulation of physiological processes in *E. coli*, and its elimination may cause diverse responses depending on the genetic background. For instance, individual deletions of *crr* or *ptsG* genes in strain W3110 have been reported to allow simultaneous consumption of xylose and glucose, with a 30% decrease in the qs_xyl_ compared to when solely xylose was used (Liang et al. 2015). While combining both deletions (Δ*crr* and *ΔptsG*) in strain C did not strongly affect qs_xyl_ (Xia et al. 2012), the same genotype applied to BL21(DE3) negatively affected the xylose growth in the xylose-glucose mixture.

Previous studies in other *E. coli* lineages suggested that both genotypes tested in ES02 and ES03 should result in strains unable to consume glucose without CCR (Eiteman et al. 2008; Xia et al. 2012). However, our results revealed that ES02 and ES03 strains were susceptible to certain levels of CCR when cultured in xylose-glucose mixtures. These findings highlight the underlying differences between *E. coli* B, C, and K-12 strains and the need for a deeper understanding of the reconfiguration of the regulatory networks in the carbon-selective engineered strains.

### The decrease in xylose consumption in the strain ES02 was alleviated by expressing a mutated version of the activator XylR^Q31K^

Xylose assimilation in *E. coli* comprises two stages, transport through the ABC transporter XylFGH or the XylE symporter (or other promiscuous transporters) followed by catabolism. In this stage, the xylose isomerase (XylA) transforms xylose into xylulose, which is further phosphorylated by the xylulose kinase (XylB) and incorporated into the pentose phosphate pathway (Zhao et al. 2020). The expression of all these genes is regulated by the activator XylR, which in turn depends on the availability of the CRP-cAMP complex (Barthe et al. 2020; Ireland et al. 2020; Heo et al. 2021).

As previously discussed, the decrease in qs_xyl_ observed in strain ES02 might be related to (i) a reduction in consumption of xylose caused by misfunction of XylFGH or (ii) weak activation of xylose catabolism caused by CCR through transcriptional regulation.

To explore the limitations in xylose consumption observed in ES02, the variant GatC ^S184L R216C^ was expressed. The expression of the variant GatC^S184L^ in a genetic background derived from K-12 promoted a twofold increase of qs_xyl_ (Utrilla et al. 2012). However, the expression of GatC ^S184L R216C^ in ES02 did not increase qs_xyl_ in either carbon source, xylose, or the xylose-glucose mixture.

Regarding transcriptional regulation, the mutated version of the xylose activator XylR^Q31K^ was expressed in strain ES02. This variant, insensitive to cAMP, was recently identified from an ALE with BL21 (DE3), resulting in a strain free of CCR that co-consumed glucose and xylose (Heo et al. 2021). The expression of XylR^Q31K^ in ES02 not only improved qs_xyl_ by 125% in cultures with xylose as the sole carbon source but also the µ_xyl_ by 150% and qs_xyl_ by 190% in mixed sugars, which agrees with previous reports (Heo et al. 2021).

The strain ES04 released CCR by the expression XylR^Q31K^, suggesting that the low qs_xyl_ in strain ES02 was caused by low levels of XylR activator, which were insufficient to fully induce the expression of the genes involved in the consumption and catabolism of xylose. Further transcriptomic analysis of ES04 grown on xylose revealed that XylR^Q31K^ mainly affected the catabolic genes *xylA* and *xylB* which presented 5.5-fold and 1.9-fold overexpression compared to BL21 (DE3)*.* Interestingly, no significant overexpression of the genes related to the xylose internalization was detected.

It was proposed that the mutation Q31K enhanced the XylR dimerization, promoting the xylose loci upregulation (Heo et al. 2021). Other XylR variants (P363S and R121C) insensitive to cAMP have also been reported in ALE experiments with W and C strains. In these strains, *xylAB* and *xylFGH* were overexpressed 10- to 20-fold, resulting in a 40 to 60% increase in the qs_xyl_ compared to the WT strain. These variants also enabled the simultaneous co-consumption of the glucose and xylose mixture. This upregulation was associated with a structural change in XylR which increased the affinity to the DNA binding sites (3- to 14-fold lower dissociation constant), allowing a more stable initiation complex and higher transcriptional rates (Sievert et al. 2017). In a further approach, the double mutant XylR^P363S R121C^ was used to replace the wild-type XylR activator on a xylose-selective strain derived from W lineage (Δ*ptsI*, Δ*ptsG*, Δ*galP*, Δ*glk*). This strain could grow in mixtures with different ratios of glucose-xylose, demonstrating the critical role of the regulator XylR in releasing CCR (Flores et al. 2020). Other studies have also reported that the availability of XylR alleviates the CCR caused by glucose (Barthe et al. 2020) and arabinose (Groff et al. 2012) when using a mixture of sugars containing xylose. Overall, the low qs_xyl_ observed in ES02 was related to insufficient levels of active XylR, which impeded the transcriptional expression of the operons involved in xylose isomerization and xylulose phosphorylation.

### Glucose consumption in strain ES04 is dependent on a functional galactitol operon

The chromosomal expression of XylR^Q31K^ in ES04 resulted in the same kinetic features (µ_xyl_ and qs_xyl_) as the episomal expression in the strain ES02/pCL_XylR^Q31K^. However, chromosomal expression of XylR^Q31K^ caused a 37% increase in maximum biomass. It is well known that plasmid maintenance consumes significant resources from the host, which can lead to effects such as low growth rates or low cell densities (Glick 1995). The chromosomal integration of XylR^Q31K^ in the strain ES04 reduced the metabolic burden, allocating more resources for biomass synthesis.

Strain ES04 outperformed BL21 (DE3), showing an improvement of 16% in µ_xyl_ and 90% in qs_xyl_ when xylose was the sole carbon source. These results agree with the enhancement reported in the ALE experiment where XylR^Q31K^ was isolated. The evolved strain JH001 showed a qs_xyl_ 50% higher than the parental BL21 (DE3) (Heo et al. 2021). XylR^Q31K^ expression alleviates the CCR observed in ES02. However, XylR^Q31K^ expression not only enhanced xylose metabolism but also stimulated the assimilation of glucose. While ES02 barely consumed glucose, strains ES02/pCL_XylR^Q31K^ and ES04 slightly recovered the ability to consume this hexose.

Transcriptomics analysis of strain ES04 grown on glucose revealed upregulation of the galactitol pathway. Further elimination of the entire operon *gatDCBAZY* (strain ES06), but not solely *gatC* (strain ES05), reduced glucose assimilation. Interestingly, the same operon was downregulated in the strain ES04 grown on xylose. The mechanism by which XylR^Q31K^ regulates the expression of *gatDCBAZY* operon in ES04 remains unknown but seems to depend on the available carbon source.

### XylR^Q31K^ expression and PTS-related genes inactivation triggered acetate accumulation in the xylose-utilizing strains

Despite similar growth rates, strains expressing XylR^Q31K^ (ES02/pCL_XylR^Q31K^ and ES04) exhibit higher xylose consumption rates (Fig. 5) and accumulate more acetate (Fig. S5) compared to strains lacking this mutated regulator (BL21 (DE3), ES02, and ES02/pCL_gatC^S184L, R216C^). This likely reflects overflow metabolism, where rapid xylose consumption causes an imbalance between acetyl-CoA production and assimilation, leading to acetate accumulation (Millard et al. 2021). Additionally, acetate production may arise from stress responses associated to PTS gene deletions. For instance, ES02 produces more acetate than BL21 (DE3) despite a lower xylose consumption rate. Deleting *gatC* in ES04 results in a 1.7-fold in acetate production in ES05, while deleting the galactitol operon results in a 2.62-fold in acetate production in ES06 (Fig. S5), even though xylose consumption rates remain comparable across strains (Fig. 5).

These different patterns in acetate accumulation suggest significant differences in the carbon distribution between biomass, acetate, and CO_2_ among strains. For instance, the biomass formation of BL21 (DE3), ES04, and ES06 were 0.89 g_DCW_/L, 0.69 g_DCW_/L, and 0.41 g_DCW_/L, respectively, the same strains directed 2.8%, 9.25, and 30% of carbon towards acetate. While deleting PTS-related genes is necessary to create a xylose-selective strain and the expression of XylR^Q31K^ improved xylose consumption to achieve the desired phenotype lacking CCR, these modifications also increase acetate accumulation. These side effects may have implications in different potential applications and should be considered when constructing xylose-selective strains.

### Xylose selectivity in* E. coli* BL21 (DE3)

The results obtained in this study show that the metabolic engineering strategies for obtaining a xylose-selective *E. coli* must be adapted to the lineage of each specific strain. This study demonstrated that applying the previously reported metabolic engineering strategies for *E. coli* K-12 and C does not produce a xylose-selective strain from the BL21 (DE3) lineage. Instead, these modifications led to an unexpected reduction in µ_xyl_ and qs_xyl_ when strains ES02 and ES03 were cultured in a xylose-glucose mixture. From the physiological behavior of ES02 in the mixture of xylose-glucose, it was proposed that XylFGH does not work correctly in BL21 (DE3) because of the truncation of XylG, leading to XylE as the main pathway for xylose consumption, where glucose might act as a competitive inhibitor. However, no improvement in qs_Xyl_ was obtained when the non-canonical xylose transporter GatC^S184L R216C^ isolated from a K-12-derived strain was expressed. Suggesting that the reduction in qs_xyl_ observed in the xylose-selective strains derived from BL21 (DE3) was not primarily related to xylose internalization capacity but rather to CCR interfering with the activation of xylose catabolism. The introduction of the XylR^Q31K^ variant, which is insensitive to cAMP, enhanced µ_xyl_ and qs_xyl_ either in xylose alone or in the mixture with glucose, allowing an enhanced use of xylose in the strain ES04 with a specific xylose consumption rate of 75% higher than that of the wild-type strain BL21 (DE3). Remarkably, the expression of XylR^Q31K^ not only improved xylose metabolism but also partially restored glucose consumption, highlighting that glucose was not consumed while xylose was present. Once xylose is exhausted, effective glucose consumption slowly initiates after a lag phase, revealing an altered CCR. The resulting strain ES04 is the first strain of *E. coli* that preferentially uses xylose over glucose.

## Supplementary Information

Below is the link to the electronic supplementary material.

Supplementary file1 (PDF 403 KB)

## Data Availability

The datasets generated during and/or analyzed during the current study are available from the corresponding author upon request.
