# Supplementary material for: Tailoring Escherichia coli BL21 (DE3) for preferential xylose utilization via metabolic and regulatory engineering
Source: Appl Microbiol Biotechnol. 2025 Feb 28;109:54. doi: 10.1007/s00253-025-13430-4 (PMC11870883; doi:10.1007/s00253-025-13430-4)
Supplement: Supplementary file 1 [file 253_2025_13430_MOESM1_ESM.pdf]

Applied Microbiology and Biotechnology

Tailoring *Escherichia coli* BL21(DE3) for preferential xylose utilization via metabolic and regulatory engineering

Eliseo R. Molina-Vázquez, Luis Caspeta, Guillermo Gosset, Alfredo Martínez *

Departamento de Ingeniería Celular y Biocatálisis, Instituto de Biotecnología, Universidad Nacional Autónoma de México. Av. Universidad 2001, Col. Chamilpa, Cuernavaca, Morelos 62210, México.

* Corresponding author:

AM: ORCID ID: 0000-0002-4804-6687; alfredo@ibt.unam.mx

Tel.: (+52) 777 3291601

emails

ERM-V: ORCID ID: 0009-0002-4021-7936; eliseo.molina@ibt.unam.mx

LC: ORCID ID: 0000-0003-4622-5179; luis.caspeta@ibt.unam.mx

GG: ORCID ID: 0000-0003-3127-0911; guillermo.gosset@ibt.unam.mx

Supplementary Information

Supplementary Table S1

Oligonucleótido	Secuencia (5’- 3’)	Descripción
F_don_crr	TTG CTG GGG ATG GGT CTG GA	Amplify the kanamycin resistance cassette flanked by FRT sequences from the genomic DNA of the strain Keio Δ*crr*, generating overhangs with homology to 226 pb upstream and 150 pb downstream *crr locus*. The amplicon was utilized for gene inactivation using the lambda red system protocol.
R_don_crr	TTA ACC GAT GCA GTG CAC CG	
F_don_xylR	ATG TCG AAA AGC AAT CCG TC	Amplify the kanamycin resistance cassette flanked by FRT sequences from the genomic DNA of the strain Keio Δ*xylR*, generating overhangs with homology to 1249 pb upstream and 1014 pb downstream *xylR locus*. The amplicon was utilized for gene inactivation using the lambda red system protocol.
R_don_xylR	TTG ACT CCC ATA CGA CGA TA	
F1_xylR	CAA TTT CAC ACA GGA AAC AGC CAT GTT TAC TAA ACG TCA CCG	Amplify the region from 1 to 106 pb of *xylR* gene, introducing the punctual mutation C91A (indicated in bold) and generating an overhang with homology to the linearized pCL1920 vector (underlined sequence).
R1_mutxylR	CCG ATT GTG ACG CCT **T**TA AAT ATT CCC CTA CGC CTT	
F2_mutxylR	AAG GCG TAG GGG AAT ATT TA**A** AGG CGT CAC AAT CGG	Amplify the region from 71 to 1,201 pb of *xylR* gene, introducing the punctual mutation C91A (indicated in bold) and generating an overhang with homology to the linearized pCL1920 vector (underlined sequence).
R2_XylR	CCT CTA GAG TCG ACC TGC AGC TAC AAC ATG ACC TCG CTA T	
F_lin_pCL	CTG CAG GTC GAC TCT AGA GG	Linearize the pCL1920 vector to facilitate circular polymerase extension cloning.
R_lin_pCL	GGC TGT TTC CTG TGT GAA ATT G	

Table S1 List of primers utilized in the molecular biology procedures for constructing the strains and plasmids

Supplementary Figure S1

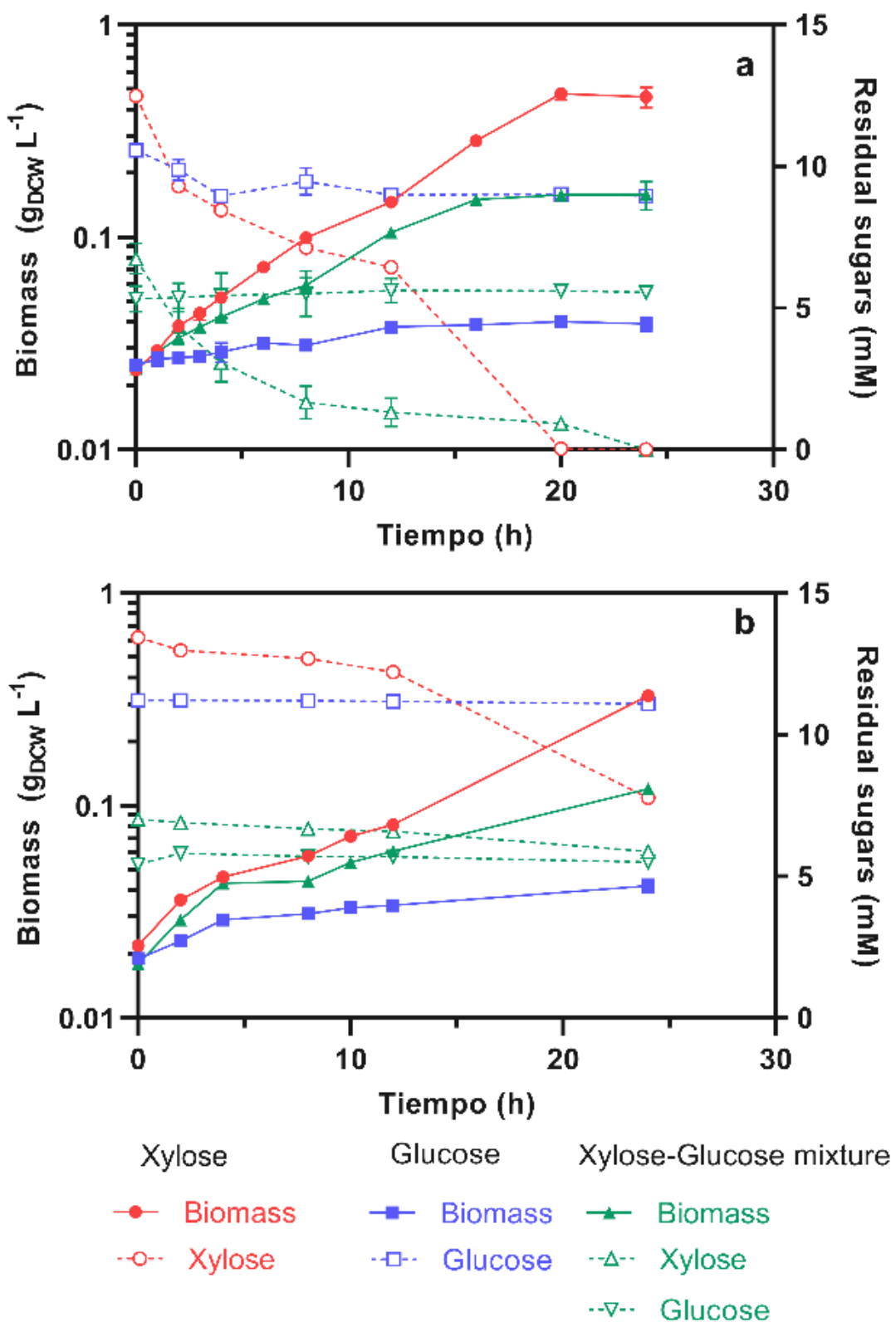

Figure S1 Growth and residual sugars kinetics of the strains a) ES02 (BL21 DE3 Δ*glk*, Δ*manZ*, Δ*ptsG*) and b) ES03 (BL21 DE3 Δ*glk*, Δ*manZ*, Δ*ptsG*, Δ*crr*) in defined M9 medium with 16 mM xylose (circles) or 14 mM glucose (squares), or a mixture (triangles) of 8 mM xylose with 7 mM glucose.

Supplementary Figure S2

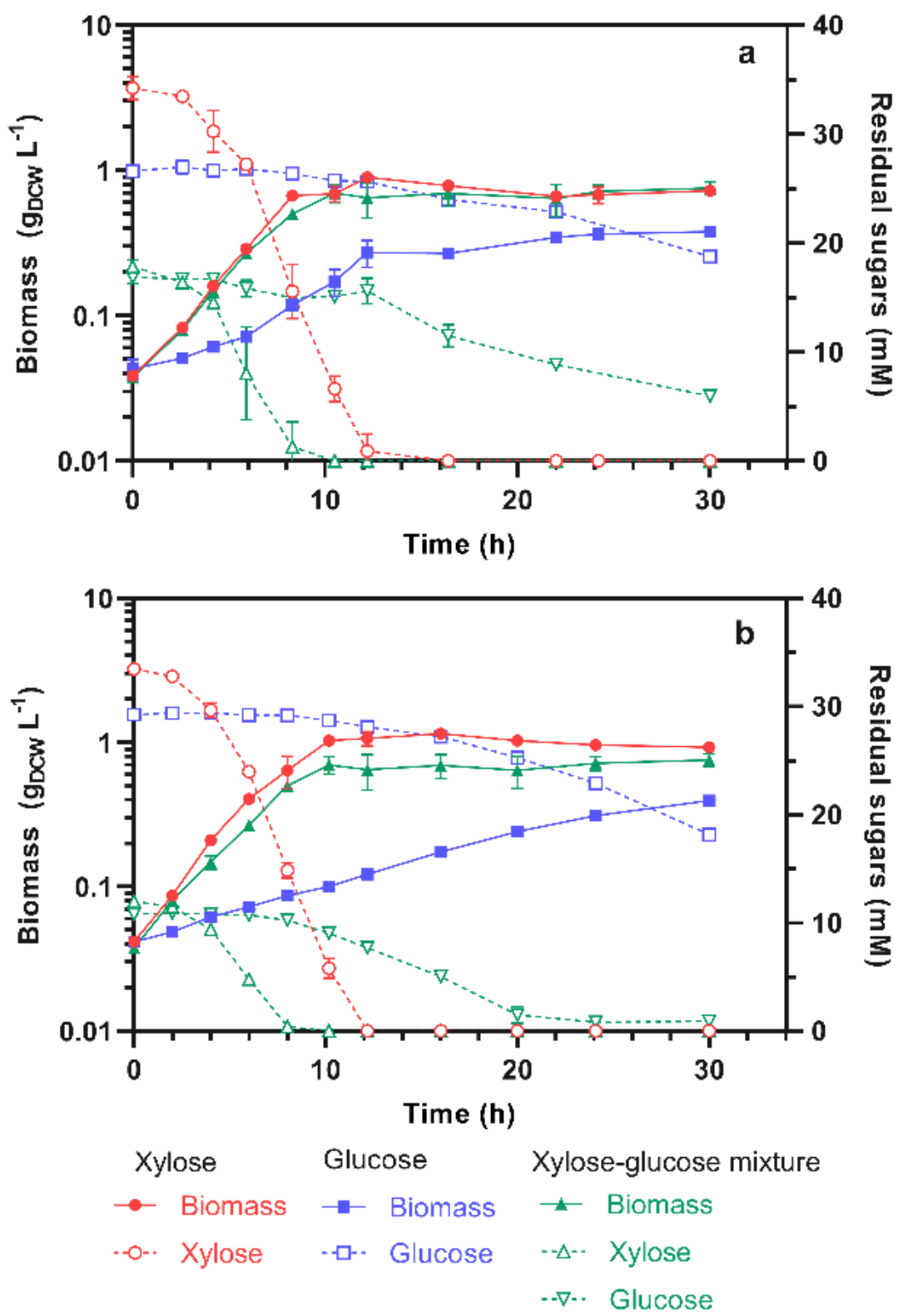

Figure S2 Growth and residual sugars kinetics of the strains a) ES04 (ES02 *xylR*::*Km*^{r}, *lacZ*::*xylR*^{C91A}-*Gm*^{r}) and ES05 (ES02 Δ*xylR*, *lacZ*::*xylR*^{C91A}-*Gm*^{r} *gatC*::*Km*^{r}) in defined M9 medium with 33 mM xylose (circles) or 28 mM glucose (squares), or a mixture (triangles) of 16.5 mM xylose with 14 mM glucose.

Supplementary Figure S3

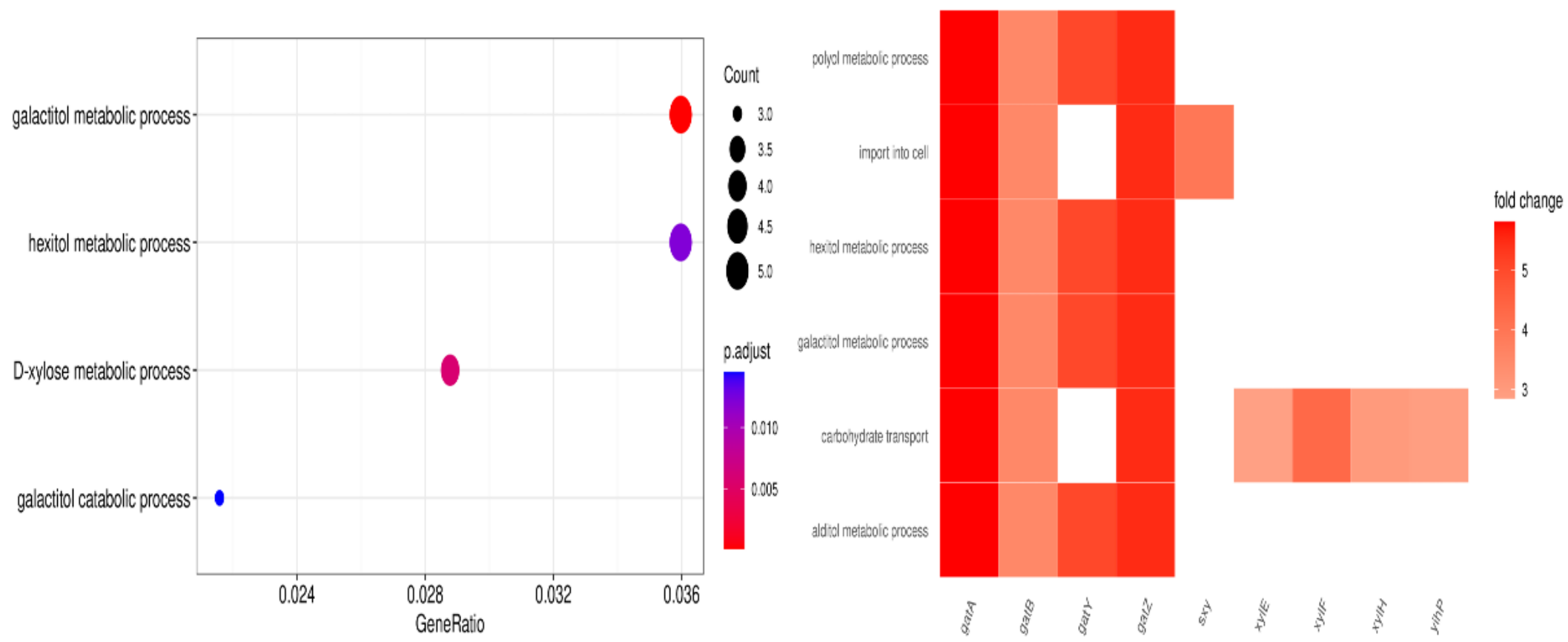

Figure S3 Dot and heat map resulting from Gene Set Enrichment Analysis (GSEA) from ES04 compared to BL21(DE3) strains cultured in glucose. Differentially expressed genes were selected by calculating an intensity-dependent Z-score as $zi = (Ri - mean(R))/(sd(R))$, where zi is the Z-score for each element gene, Ri is the log-ratio for each element gene, and sd(R) is the standard deviation of the log-ratio. Genes with |z-score| > 2 standard deviations were considered significantly expressed genes. The analysis was performed by ClusterProfiler package, which was used to perform gene enrichment analysis (GEA), and pValue and qValue were adjusted by the HB method with a cutoff value <0.05

Supplementary Figure S4

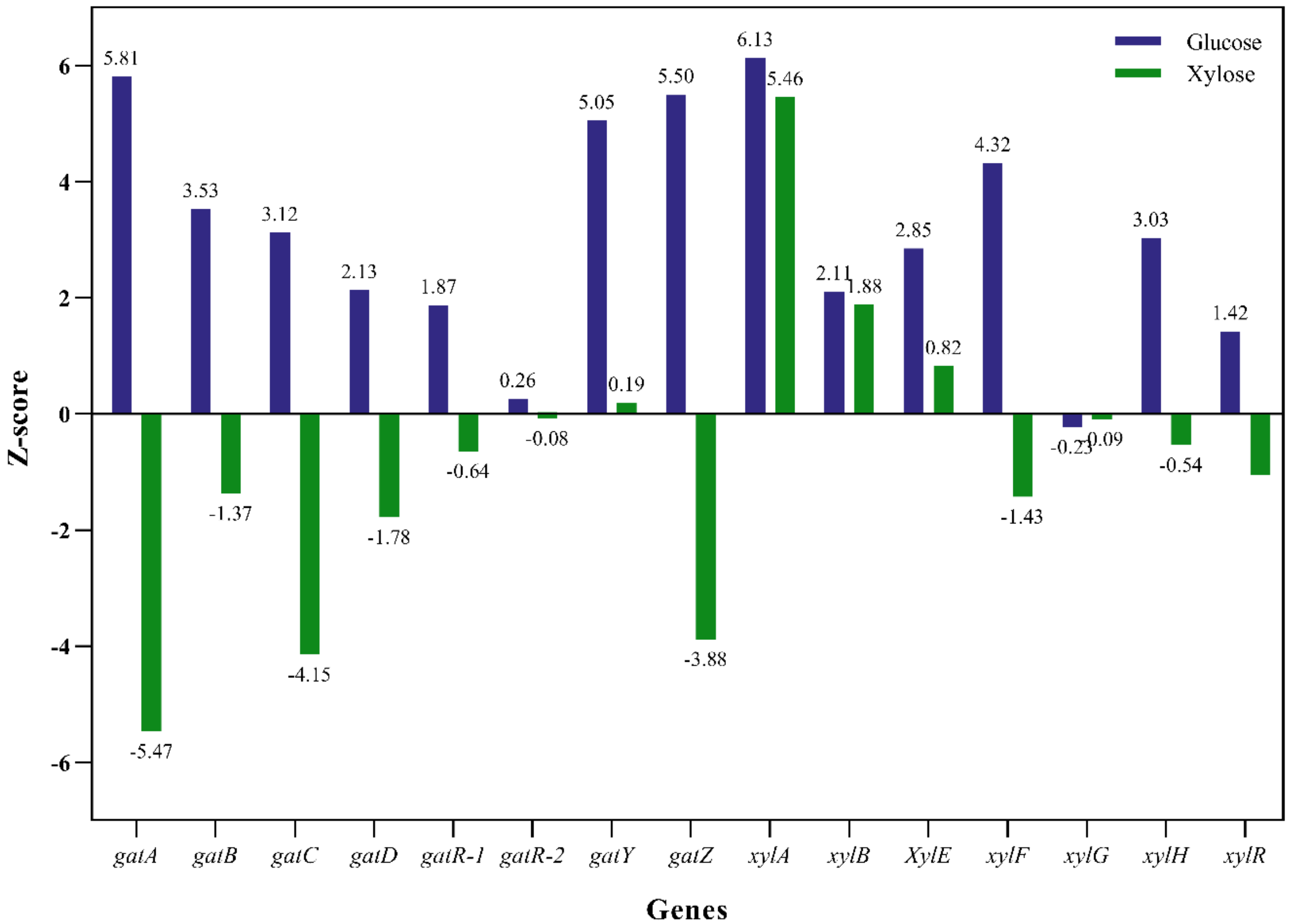

Figure S4 Differentially expressed genes of ES04 compared to BL21(DE3) with xylose and glucose. Z-score as zi = (Ri − mean(R))/(sd(R)), where zi is the z score for each element gene, Ri is the logratio for each element gene, and sd(R) is the standard deviation of the log ratio

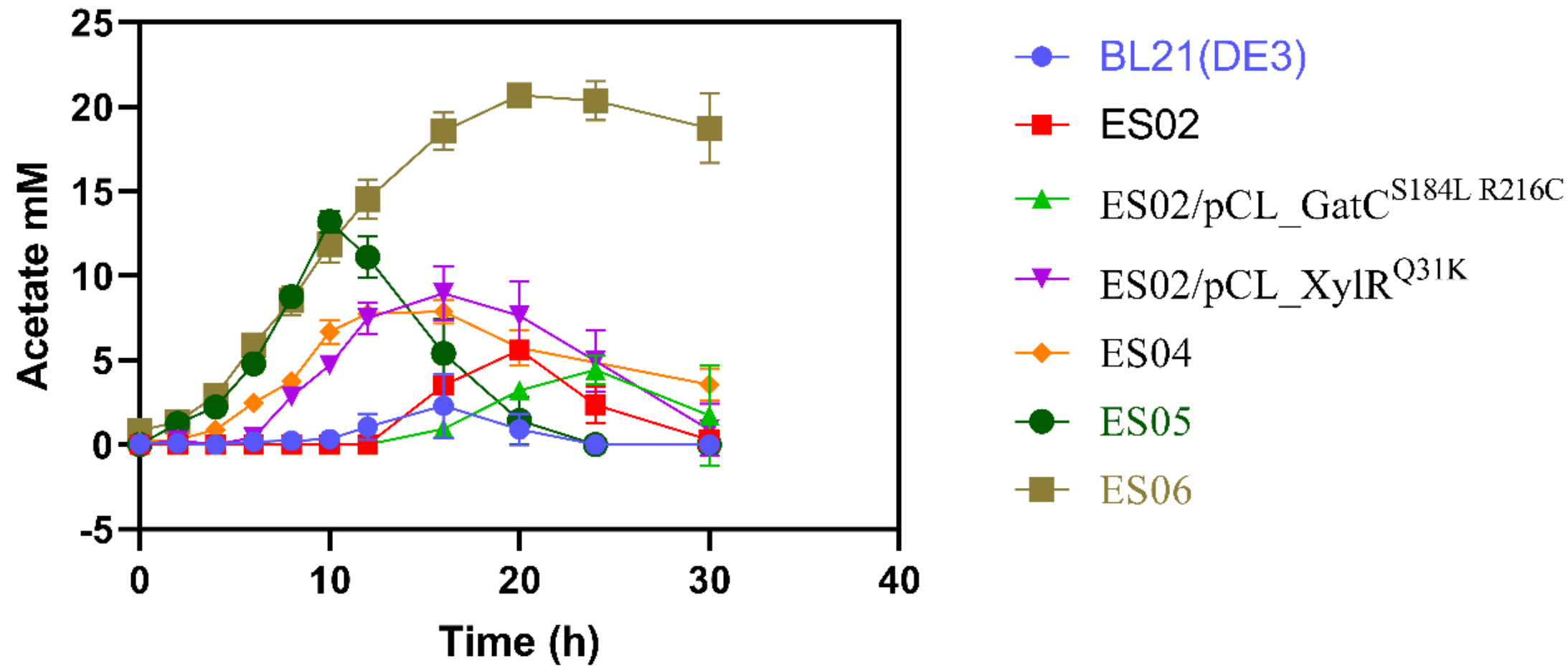

Figure S5 Acetate production kinetics of the different *E. coli* strains evaluated in this study using 33 mM xylose as carbon source.
